# Familial Mediterranean Fever-Related Peritonitis Visualized on Computed Tomography

**DOI:** 10.31662/jmaj.2025-0259

**Published:** 2025-09-12

**Authors:** Yuki Ohnishi, Keisuke Ueno, Masashi Kusakabe, Yasuhiro Suyama

**Affiliations:** 1 Department of Rheumatology, NTT Medical Center Tokyo, Tokyo, Japan; 2 Department of Surgery, Nitobe Memorial Nakano General Hospital, Tokyo, Japan; 3 Department of Radiology, NTT Medical Center Tokyo, Tokyo, Japan

**Keywords:** fever, abdominal pain, familial Mediterranean fever, computed tomography

A 33-year-old man presented to the clinic for a 5-year history of recurrent abdominal pain. The episodes occurred every 1-3 months, characterized by the simultaneous onset of fever up to 38°C and abdominal pain. Symptoms were most severe on the first day, with pain initially localized to the right lower quadrant and subsequently spreading to the entire abdomen. Fever resolved within a day, while abdominal pain subsided over 2-3 days. Laboratory investigations revealed an elevated white blood cell count of 14,800/μL and a C-reactive protein (CRP) level of 0.56 mg/dL. An enhanced computed tomography (CT) scan revealed thickening of the peritoneum on the right side of the midline in the pelvic region and increased density of adipose tissue, without abnormalities in the intestine (Figure 1), suggesting peritoneal inflammation. Genetic analysis identified a heterozygous M694I mutation in exon 10 of the MEFV gene, which is known to be responsible for familial Mediterranean fever (FMF). Colchicine alleviated his symptoms. We diagnosed FMF.

**Figure 1.**
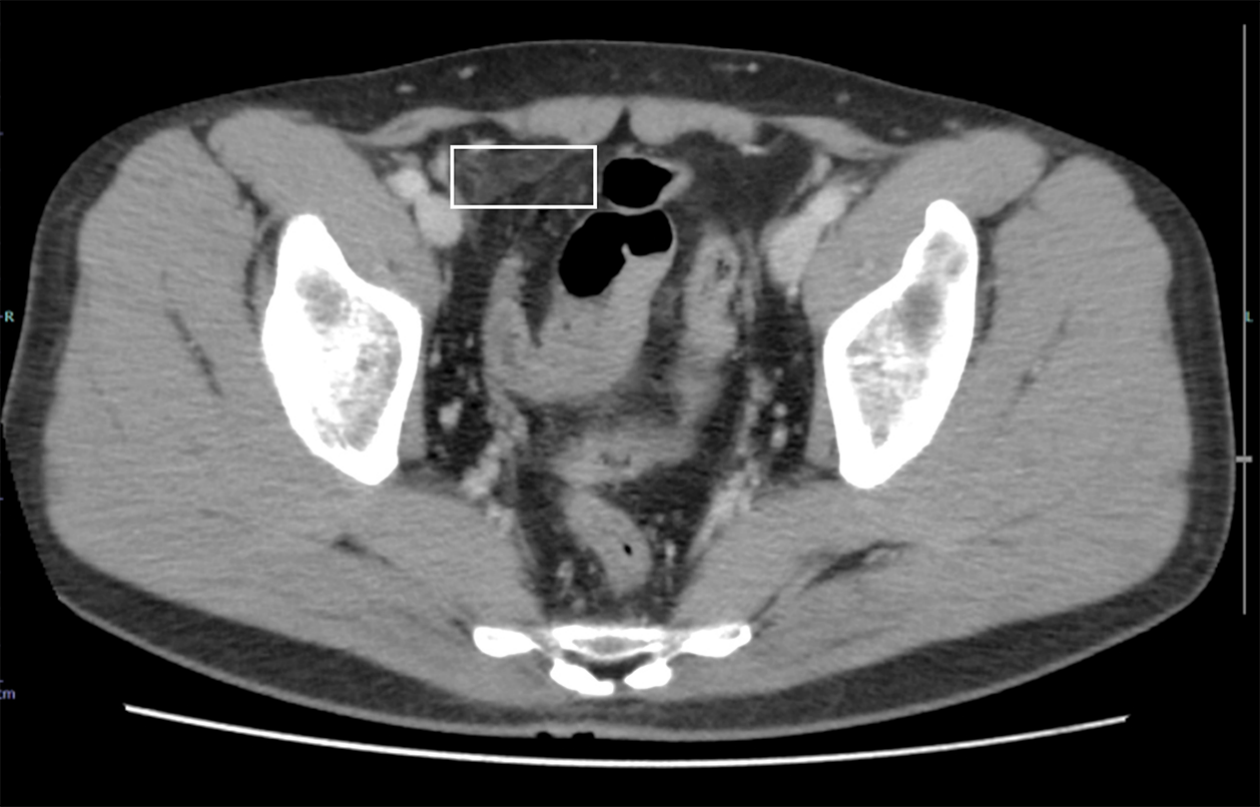
Thickening of the peritoneum along the right paramedian pelvic region with increased density of adjacent adipose tissue, without abnormal intestinal findings.

FMF should be considered in patients with recurrent febrile episodes and elevated CRP during attacks ^(1)^. Early diagnosis may be difficult before the characteristic fever pattern emerges, but abdominal CT findings―such as engorged mesenteric vessels, thickened mesenteric folds, mesenteric lymphadenopathy, ascites, focal peritonitis, dilated small bowel loops, and mural thickening of the ascending colon ^(2), (3)^―can help distinguish FMF from other causes of acute abdomen.

## Article Information

### Author Contributions

According to the definition given by the International Committee of Medical Journal Editors (ICMJE), the following individuals qualify for authorship based on their substantial contributions to the manuscript’s intellectual content: Yuki Ohnishi and Yasuhiro Suyama, conception, design, and writing of the manuscript; Masashi Kusakabe, Keisuke Ueno, and Yasuhiro Suyama, acquisition of data. All authors have read and approved the manuscript.

### Conflicts of Interest

None

### Ethics Approval and Consent to Participate

The authors obtained consent from the patients for the publication of this report, including images.
